# Exogenous Pyruvate in Defense Against Human-Exposure Toxicants: A Review of In Vitro and In Vivo Evidence

**DOI:** 10.3390/ijms26178316

**Published:** 2025-08-27

**Authors:** Iwona Zwolak

**Affiliations:** Department of Biomedicine and Environmental Research, Institute of Biological Sciences, Faculty of Medicine, The John Paul II Catholic University of Lublin, Konstantynów Ave. 1J, 20-708 Lublin, Poland; iwona.zwolak@kul.pl

**Keywords:** pyruvate, oxidative stress, antioxidant, mitochondria, NF-κB, inflammation

## Abstract

Pyruvate is an alpha-keto acid that occurs naturally in living cells. It is a key metabolite in cellular respiration and a substrate for the synthesis of glucose (in gluconeogenesis) and certain amino acids. Exogenous pyruvate, for example in the form of sodium pyruvate or ethyl pyruvate, has potential therapeutic applications due to its antioxidant and anti-inflammatory properties. This review summarises cell culture and animal studies that report the cytoprotective effects of exogenous pyruvate compounds during exposure to environmental pollutants, drugs, UV radiation, and burns. These reports show that the main mechanisms through which exogenous pyruvate exerts its beneficial effects are the neutralisation of reactive oxygen species, protection and stabilisation of mitochondria, maintenance of ATP levels, and inhibition of inflammatory signalling pathways, including the nuclear factor-kappa B (NF-κB) pathway. The article also outlines potential challenges associated with the therapeutic use of exogenous pyruvate. These include the instability of inorganic pyruvate (sodium pyruvate) and the fact that the metabolism of ethyl pyruvate differs between humans and animals.

## 1. Introduction

**Figure 1:**
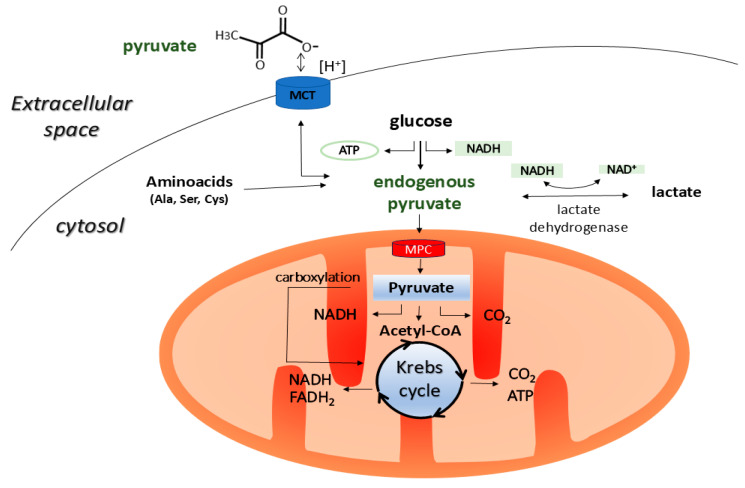
Pyruvate is mainly produced in the cytoplasm of animal cells by glycolysis. It can also be formed in the cytoplasm through the metabolism of malate, lactate, and amino acids such as alanine, cysteine, serine, and glycine. The resulting pyruvate is then transported to the mitochondria via the MPC, which is located in the inner mitochondrial membrane, where it is incorporated into the Krebs cycle by being converted into acetyl-CoA. In addition, pyruvate can undergo carboxylation in the mitochondria to form oxaloacetate, which is a common intermediate in both the Krebs cycle and gluconeogenesis. In the cytoplasm, pyruvate can also be converted into lactate under oxygen-limited conditions, or it can be used for synthesising alanine or secreted from the cell into the intercellular space via the MCT transporter in symport with H^+^ [1,2]. MCT, monocarboxylate transporter; MPC, mitochondrial pyruvate carrier; NAD^+^, oxidized nicotinamide adenine dinucleotide; NADH, reduced nicotinamide adenine dinucleotide.

Pyruvate (CH_3_COCOO^−^) is a simple three-carbon α-keto acid that is produced naturally in all living cells. It is primarily a product of glycolysis and an important intermediate in glucose metabolism and cellular energy production. Endogenous pyruvate, which is produced by glycolysis, enters the mitochondria, where it either feeds the Krebs cycle or is converted into lactate during periods of oxygen deprivation. It can also be used in the synthesis of alanine or to support gluconeogenesis [1,2] (Figure 1).

**Figure 2:**
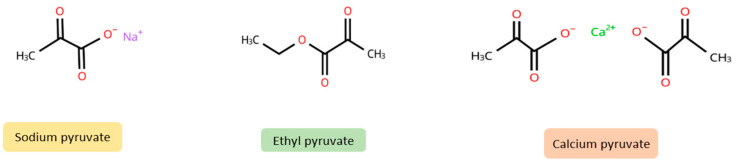
Chemical structures of sodium pyruvate, ethyl pyruvate, and calcium pyruvate (created using chemspider) (https://www.chemspider.com/StructureSearch, accessed on 15 April 2025).

Furthermore, exogenous pyruvate (see Figure 2 for chemical structures), mainly in the form of ethyl pyruvate and sodium pyruvate supplements, is known for its antioxidant and anti-inflammatory properties [1,3]. Small amounts of pyruvate can also be found in food. Foods rich in pyruvate include milk (0.303 mM), red wine (0.446 mM), white wine (0.139 mM), and apple juice (0.75 mM) [4]. The beneficial effects of exogenous pyruvate have been demonstrated in animal models of phenylketonuria [5], epilepsy [6], and burns [7,8], among others. In human studies, inhaled pyruvate improved parameters in patients with COPD [9,10] and in patients with COVID-19 [11], while oral or intravenous pyruvate had beneficial effects in patients with mitochondrial diseases [12], liver disease [13], diabetes [14], and glaucoma [15]. In addition, pyruvate compounds, which include both organic and inorganic derivatives, have a variety of applications in areas such as cosmetics, food production, and the chemical industry (see Table 1). Pyruvate is also an optional ingredient in culture media used for growing cell lines in vitro, where it serves as an additional energy substrate. Depending on the formulation, culture media typically contain 0.5–1 mM (55–110 mg/L) of sodium pyruvate.

The first reports on the cytoprotective and antioxidant properties of exogenous pyruvate in cultured mammalian cells were published in 1985 [24] and 1987 [25]. These studies demonstrated the ability of pyruvate to act as an antioxidant against hydrogen peroxide (H_2_O_2_)-induced cytotoxicity in cultures of the Chinese hamster V79 cell line, as well as in mouse cell lines (P388 lymphoma and P815 mastocytoma). These reports also suggested that pyruvate may have a similar protective function against H_2_O_2_ generated in blood plasma in vivo [24,25]. Subsequent studies in experimental models in vitro [26,27,28] and in vivo [29] have confirmed pyruvate’s protective capacity against pro-oxidant conditions in cells and tissues. Since then, studies describing the antioxidant and anti-inflammatory properties of pyruvate compounds have been published regularly.

Oxidative stress is a common pathological condition that occurs when cells and tissues are exposed to toxic agents, such as UV radiation or environmental pollutants. This condition is characterised by the excessive production of reactive oxygen species (ROS) and a reduction in antioxidant defences, which leads to oxidative damage to cellular structures and cell death. At the same time, these changes activate pro-inflammatory pathways, including those related to nuclear factor kappa B (NF-κB). This leads to inflammation and further tissue damage, thereby promoting the development of various diseases [30,31]. It is well known that enhancing the body’s antioxidant defences protects cells exposed to toxic agents and prevents pathological changes. In this review, we will: (1) summarise preclinical studies (in vivo and in vitro) that highlight the protective effects of exogenous pyruvate against damage induced by physical and chemical toxicants; (2) discuss the antioxidant and anti-inflammatory mechanisms involved in pyruvate’s cytoprotective effects; (3) present issues that hinder the use of exogenous pyruvate as an antioxidant.

## 2. Pyruvate Effects on Physical and Chemical Toxicants

### 2.1. In Vivo Studies

Table 2 summarises the results of 24 studies that investigated the effects of exogenous pyruvate against toxic agents in vivo. Most of these studies used rat or mouse models. One study used guinea pigs as test subjects. The form of exogenous pyruvate primarily studied was ethyl pyruvate, which was administered to the animals in doses ranging from 40 to 100 mg/kg. In addition, some researchers analysed the effects of aromatic pyruvate derivatives [32] and sodium pyruvate [33]. Two papers did not specify the form of pyruvate used in the study [34,35]. Most of these studies found that the administration of exogenous pyruvate significantly reduced the adverse health effects caused by heat stress [36], ionising radiation [37,38], UV radiation [34], analgesics [39], chemotherapeutic agents [40,41], alcohol [42] as well as other substances. The protective effects of pyruvate have been observed in various organs of animals, including the skin, lungs, liver, and brain. These effects included the normalisation of parameters related to oxidative stress, such as reduced lipid peroxidation and increased antioxidant defences [43,44]. Anti-apoptotic and anti-inflammatory effects were also observed, including inhibition of necrotic lesions and release of pro-inflammatory factors from tissues [36,38,45]. The signalling pathways affected by pyruvate included those related to the molecules NF-κB, Toll-like receptor 4 (TLR4), and High Mobility Group Box 1 (HMGB1) [36,43,46].

### 2.2. In Vitro Studies

Table 3 lists in vitro studies investigating the effectiveness of pyruvate in combatting physical and chemical cytotoxicity in various animal cell models. The in vitro models employed in these studies included RAW264.7 macrophages [38,53], human fibroblasts [56], human bronchial epithelial cells [57], and primary neuronal cell lines [58]. Studies have mostly used sodium pyruvate or ethyl pyruvate at millimolar concentrations, with some studies using ethyl pyruvate at micromolar doses. In studies, pyruvate showed protective effects against toxic effects induced by radiation (ionising and UV) [34,37], H_2_O_2_ [59], metals (Cd, V, Al, Zn) [43,60,61,62], paracetamol metabolites [39], organic dusts, and cigarette smoke [57,63]. The actions of pyruvate included antioxidant effects (reduction of ROS levels, increase in GSH levels) [63,64], stabilisation of mitochondrial function [61], increase in ATP levels [26], increase in expression of proteins associated with the Nrf2 pathway [63], reduction of inflammatory mediators (e.g., HMGB1, NF-κB) [38,57], anti-apoptotic, and anti-necrotic effects [39].

## 3. Cytoprotective Modes Activated by Exogenous Pyruvate in Animal Cells

### 3.1. Direct ROS Scavenging Activity

Alpha-keto acids undergo rapid decarboxylation in the presence of H_2_O_2_, resulting in the formation of a carboxylic acid, water, and carbon dioxide according to the reaction: R-COCOOH + H_2_O_2_ → R-COOH + H_2_O + CO_2_. First described in 1904, this reaction is the primary mechanism responsible for the antioxidant (reactive oxygen species (ROS) scavenging) properties of pyruvate compounds. Due to the relatively high concentration of pyruvate in tissues (in the micromolar range), early reports considered it to be one of the main elements of the cellular antioxidant defence, alongside catalase and glutathione peroxidase, which regulate the level of H_2_O_2_ in the cellular environment. One clear advantage of the reaction of pyruvate with H_2_O_2_ over enzymatic reactions for neutralising H_2_O_2_ is that non-enzymatic decarboxylation of pyruvate does not involve the formation of toxic by-products [67,68]. Furthermore, the scavenging activities of ethyl pyruvate and sodium pyruvate against superoxide anion and hypochlorous acid were detected using chemiluminescence and spectrophotometry, respectively [55,69]. Vasquez-Vivar et al. [70] found that pyruvate can also react with peroxynitrite according to the scheme CH_3_COCOO^−^ + ONOO^−^ → CH_3_COO^−^ + NO_2_^−^ + CO_2_. This reaction proceeds with an apparent second-order rate constant of 88 M^−1^ s^−1^ and is one order faster than the reaction of pyruvate with H_2_O_2_ (2.2 M^−1^ s^−1^) [70]. Nagatome et al. [39] also reported the ability of 1 and 10 mM ethyl pyruvate to scavenge peroxynitrite, using dihydrorhodamine as a peroxynitrite activity probe. The ability of ethyl pyruvate to scavenge free radicals was confirmed using 2,2-diphenyl-1-picrylhydrazyl (DPPH) and ferric reducing antioxidant power (FRAP) assays [71]. In these assays, the ability of ethyl pyruvate to scavenge the synthetic DPPH radical and reduce Fe^3+^ to Fe^2+^ ions (FRAP assay) increased proportionally with the concentration of ethyl pyruvate [71].

As mentioned above, pyruvate can be considered part of the cellular antioxidant defence system because it can directly neutralise H_2_O_2_ (and other ROS). However, according to a study by Guarino et al. [72], the role of intracellular pyruvate in H_2_O_2_ elimination may be insignificant. In fact, the authors of the study calculated that the elimination time of intracellular H_2_O_2_ in the presence of physiological levels of pyruvate is far too long (95% of 1 µM H_2_O_2_ is removed in approximately 150 min), compared to the rate at which H_2_O_2_ is neutralised by cytosolic glutathione peroxidase (95% of 1 µM H_2_O_2_ is removed in just 0.06 s). However, it was noted that pyruvate may play an important role in the elimination of H_2_O_2_ in extracellular spaces where the concentration of H_2_O_2_ is much higher and peroxidase activity is minimal, considering that pyruvate concentrations can rise to 1 mM [72]. Approximately, this concentration of pyruvate, i.e., 0.84 mM, was physiologically achievable in the blood of human volunteers 20 min after intravenous administration of sodium pyruvate [73].

### 3.2. Effects on Mitochondrial Functions

In vitro studies of this α-ketoacid have reported beneficial effects of pyruvate on mitochondrial function, such as mitochondrial membrane potential, against chemical and physical agents [37,60,61,63,74]. In support of this, research by Kang et al. [75] on cultured bovine pulmonary artery endothelial cells showed that blocking the transport of pyruvate into the mitochondria using the specific inhibitor α-cyano-3-hydroxycinnamate significantly reduced pyruvate’s protective effect against the pro-apoptotic properties of H_2_O_2_. This suggests that pyruvate metabolism within the mitochondria is involved in the cytoprotective effects of this compound.

Pyruvate affects mitochondria in several ways. Firstly, it stimulates pyruvate dehydrogenase activity by strongly inhibiting pyruvate dehydrogenase kinase. This increases the conversion of pyruvate to acetyl-CoA, which is a key molecule in the mitochondrial Krebs cycle [76]. Secondly, pyruvate can replenish Krebs cycle intermediates by converting directly to oxaloacetate in a reaction catalysed by pyruvate carboxylase [76,77]. This helps to maintain the Krebs cycle and the production of NADH and FADH, which are required for oxidative phosphorylation and ATP synthesis. Additionally, NADH derived from the Krebs cycle can also transfer electrons to nicotinamide adenine dinucleotide phosphate (NADP) via nicotinamide nucleotide transhydrogenase (NNT), which is a protein located in the inner mitochondrial membrane. The NADPH, via the activities of glutathione reductase and thioredoxin reductase, is used to reconstitute cellular reductants, i.e., reduced glutathione (GSH) and thioredoxin (Trx), respectively. This enhances mitochondrial antioxidant defences [78,79]. It is worth noting that clinical studies in people with incurable mitochondrial diseases also suggest that exogenous pyruvate plays a supportive role in maintaining mitochondrial function. Administration of pyruvate to patients alleviated some of the symptoms of these diseases [1].

In addition, cells can use exogenous pyruvate to synthesise aspartate, bypassing the mitochondrial-dependent synthesis of this amino acid. This is particularly important when the electron transport chain (ETC) in the mitochondria is damaged, as this chain plays an essential role in the synthesis of aspartate. Notably, aspartate is essential for cellular protein, purine, and pyrimidine synthesis. Therefore, inhibition of aspartate synthesis by impairment of ETC inhibits cell proliferation. When mitochondrial function related to the electron transport chain is impaired, pyruvate supplementation allows mitochondrial-independent synthesis of aspartate and preservation of cell proliferation despite mitochondrial damage [80,81]. In this way, exogenous pyruvate may protect the ability of cells to proliferate in the presence of mitochondrial toxic agents, such as mitotoxic antibiotics [82].

### 3.3. Anti-Inflammatory Activity of Pyruvate

Inflammation is the body’s defence response to a toxic agent of biological, chemical, or physical origin. However, chronic and excessive inflammation can exacerbate cell and tissue damage. Various in vitro and in vivo studies, summarised in Table 2 and Table 3, have reported that pyruvate inhibits the release of inflammation-related cytokines, such as COX2, HMGB1, and TNF-α, under conditions of physical and chemical challenge. The anti-inflammatory properties of pyruvate are probably partly due to its antioxidant effects, as oxidative stress and inflammation are known to be mutually supportive. However, as Fink [83] points out, some in vitro studies have shown that ethyl pyruvate exhibits stronger anti-inflammatory effects than known antioxidants (e.g., N-acetylcysteine). This suggests that the anti-inflammatory properties of ethyl pyruvate are not solely due to ROS scavenging. Therefore, below is a brief summary of main anti-inflammatory mechanisms activated by pyruvate, which may contribute to its cytoprotective effects.

#### 3.3.1. Reduction of NF-κB Activity

Nuclear factor-κB (NF-κB) is a family of inducible transcription factors that occur as homodimers or heterodimers of Rel/NF-κB family proteins (p50/p105, p52/p100, RelB, c-Rel, and p65). NF-κB is found in the cytoplasm of animal cells in an inactive form, associated with inhibitory IκB proteins. The NF-κB proteins are released when activated by stress factors such as cytokines, UV irradiation, cigarette smoke, and heavy metals. They then translocate to the nucleus, where they regulate the transcription of genes associated with the inflammatory response [84]. Some studies suggest that the inhibition of the NF-κB pathway is important for the anti-inflammatory mechanism of exogenous pyruvate when the body is exposed to toxic chemicals or physical agents [36,46,57]. Pyruvate may act on the NF-κB pathway by a variety of mechanisms. Hu et al. [36] reported that the inhibitory effect of pyruvate on NF-κB activation induced by heat stroke (as measured by p65 phosphorylation) in rat livers is mediated by the induction of the antioxidant enzyme heme oxygenase-1 (HO-1) and the preservation of autophagy. Furthermore, another proposed mechanism may be the induction of heat shock protein (HSP) 70 synthesis by pyruvate. This can inhibit the phosphorylation and degradation of IκB, thus blocking the activation of NF-κB proteins [36]. Bhat et al. [57] observed that ethyl pyruvate blocked the translocation of NF-κB p65 from the cytoplasm to the nucleus when induced by organic dust. In addition, Han et al. [85] proposed that there is a direct interaction between pyruvate and the NF-κB factor. The researchers proposed that ethyl pyruvate inhibits NF-κB signalling in human embryonic kidney 293 cells by directly modifying the NF-κB structural protein subunit p65, thereby blocking the binding of the NF-κB factor to DNA [85].

#### 3.3.2. Blocking HMGB1 Secretion

High mobility group box 1 (HMGB1) is a nuclear protein that plays an important role in cellular processes such as transcription, DNA replication and repair, and the regulation of chromatin structure under physiological conditions. In contrast, when cellular injury occurs, HMGB1 is released into the cytoplasm and then into the extracellular environment, where it acts as a damage-associated molecular pattern molecule (DAMP). Excessive amounts of secreted HMGB1 aggravate inflammation [86,87]. Pyruvate, especially in the form of ethyl pyruvate, is a known HMGB1 antagonist and reduces HMGB1 secretion in infections and inflammatory diseases (see Yang et al. [3] for a review). Similarly, inhibition of HMGB1 by ethyl pyruvate may contribute to its anti-inflammatory and cytoprotective effects in inflammation induced by chemical or physical toxicants. As summarised in Table 2 and Table 3, ethyl pyruvate inhibits the radiation-induced release of HMGB1 into the extracellular environment in both RAW264.7 murine macrophage cells and HBE human bronchial epithelial cells [38]. Another study found that ethyl pyruvate inhibited the nuclear-to-cytoplasmic transport of HMGB1 induced by organic dust extract in an airway epithelial cell model [57] and reduced the overexpression of this protein in the liver of CCl_4_-treated rats [46].

There are several possible mechanisms by which ethyl pyruvate inhibits HMGB1 secretion. For example, Seo et al. [88] demonstrated that ethyl pyruvate inhibited ischaemia-reperfusion-related HMGB1 release from renal cells in mice by inducing heme oxygenase-1 (HO-1) expression; this effect was abolished by zinc protoporphyrin, an HO-1 inhibitor. In another study, ethyl pyruvate was found to inhibit the secretion of HMGB1 by lipopolysaccharide (LPS)-activated BV2 microglia cells. This was achieved by blocking the phosphorylation of HMGB1 induced by LPS, which is likely due to the inhibition of protein kinase C alpha (PKCα) and calcium/calmodulin-dependent protein kinase (CaMK) IV activity by ethyl pyruvate. These two enzymes are required for HMGB1 phosphorylation [89]. Another study by the same research group showed that ethyl pyruvate reduces Ca^2+^ levels in BV2 cells, presumably through chelation. This inhibits the activation of PKCα and CaMKIV kinase, as well as the phosphorylation and secretion of HMGB1 from cells [90]. In addition, other authors [91] demonstrated that ethyl pyruvate inhibited HMGB1 acetylation and secretion by LPS-stimulated RAW 264.7 macrophages by modulating the SIRT1/STAT pathway, i.e., induction of the deacetylase Sirtuin 1 (SIRT1) and inhibition of LPS-induced STAT1 protein phosphorylation.

#### 3.3.3. Nrf2 Induction

Nuclear factor erythroid 2-related factor 2 (Nrf2) is a transcription factor that plays a key role in defending cells against various stress factors, including xenobiotics, inflammation, and the accumulation of abnormal proteins. Under physiological conditions, Nrf2 is present at low levels in an inactive form in the cytoplasm, where it is constantly being degraded. However, when activated by oxidative, electrophilic or proteotoxic stress, Nrf2 evades proteasome-mediated degradation. Instead, it translocates to the nucleus, where it binds to antioxidant response element (ARE) sequences in the promoter region of certain genes, thereby stimulating their transcription. The Nrf2 factor targets genes that encode antioxidant enzymes, drug-metabolising enzymes, and metabolic enzymes [92]. Nrf2 plays a role in preventing inflammation in part by regulating the expression of genes associated with the ARE sequence [93]. Studies report that exogenous pyruvate can positively affect Nrf2 signalling [94], leading to Nrf2 activation via several mechanisms. For instance, ethyl pyruvate has been shown to induce a cytoprotective effect against H_2_O_2_ toxicity by stimulating Nrf2, which then leads to Nrf2-mediated HO-1 induction in primary cultures of astrocytes. Further analysis revealed the involvement of the protein kinase B (Akt) signalling pathway and mitogen-activated protein kinases (MAPK), including extracellular signal-regulated kinase (ERK), in ethyl pyruvate-induced Nrf2 nuclear translocation [95]. In addition, the involvement of p38 MAPK, but not ERK and Jun N-terminal kinase (JNK) MAPKs, in the ethyl pyruvate-dependent activation of Nrf2 has been reported in RAW 264.7 cells [96]. Kim et al. [97] investigated the role of the interaction of Nrf2 with its transcriptional co-activator p300 in the induction of anti-inflammatory effects by ethyl pyruvate in a BV2 microglial cell line. In this study, ethyl pyruvate was found to stimulate Nrf2 transcriptional activity by enhancing the interaction between Nrf2 and P300, while reducing the interaction between P300 and p65. This attenuation of the interaction between P300 and p65 (where P300 acts as a co-activator for p65, a NF-κB subunit) has anti-inflammatory effects [97].

### 3.4. Other Possible Protective Mechanisms of Pyruvate

Exogenous pyruvate increased intracellular citrate and isocitrate levels in mouse cortical neuronal cells while providing neuroprotection against zinc-induced cytotoxicity. In this case, pyruvate’s indirect neuroprotective mechanism was related to the ability of citrate and isocitrate to directly chelate free zinc ions, thereby reducing their neurotoxicity [58]. In addition, Kim et al. [62] suggested that the protective effect of pyruvate against zinc (Zn) toxicity in mouse cortical neurons may be related to the regeneration of nicotinamide adenine dinucleotide (NAD) through the reduction of pyruvate to lactate in a reaction catalysed by lactate dehydrogenase (LDH). They based this conclusion on the observation that inhibiting NAD synthesis through LDH-competitive inhibition reduced the protective effect of pyruvate on these cells [62]. Finally, it has been suggested that exogenous pyruvate may be effective in treating metabolic acidosis, which can occur in cases of alcohol poisoning, for example. In this case, pyruvate’s possible mechanism of action is the result of the metabolic reactions that the compound undergoes in cells. These reactions include the LDH-catalysed reduction of pyruvate to lactate, and the oxidation of pyruvate to acetyl-CoA (by pyruvate dehydrogenase), which is then converted to water and carbon dioxide in the Krebs cycle. These reactions are associated with intracellular proton consumption and the correction of cellular acidosis (see Zhou [2] for a review).

## 4. Potential Barriers to the Use of Pyruvate as a Protective Agent

The instability of pyruvate, particularly in its inorganic form (e.g., sodium pyruvate), is often cited as an argument against its use as a drug. Indeed, sodium pyruvate is unstable in solutions with a pH above 6.0 at room temperature, undergoing spontaneous aldol condensation reactions and producing a potentially toxic by-product: pyruvate dimer (parapyruvate, 4-hydroxy-4-methyl-2-ketoglutaric acid). Parapyruvate has been found in industrial sodium pyruvate preparations and commercial calcium pyruvate supplements. The latter contain parapyruvate concentrations ranging from 1.4% to 10.6%, which could potentially have harmful biological effects [98,99]. However, Zhou [100] reported that one way to prevent parapyruvate formation in aqueous sodium pyruvate solutions is to maintain a pH of around 4.5. This renders the pyruvate solution stable for two years at room temperature (U.S. patent: 8,835,508 B2, 2014). The shelf life of pyruvate solutions can also be extended by using relatively dilute solutions (lower concentrations of pyruvate slow the formation of parapyruvate). Additionally, commercially available sodium pyruvate solutions (at a concentration of 100 mM) for cell culture supplementation have a recommended storage temperature of 2–8 °C, which maintains a shelf life of 1 year [101]. Furthermore, animal studies have shown that administration of sodium pyruvate can lead to transient hypernatraemia [102]. This effect does not occur when N-1-methylnicotinamide pyruvate (MNA pyruvate) is used instead. MNA pyruvate retains the protective properties characteristic of pyruvate [103]

Some studies report that ethyl pyruvate, an aliphatic ester, derived from pyruvic acid, is more stable in aqueous solutions and therefore more protective than sodium pyruvate. Ethyl pyruvate is also more hydrophobic, which makes it more likely that it can diffuse into cells more rapidly than the pyruvate anion [104,105]. For example, Shin et al. [95] compared the protective effects of ethyl pyruvate and sodium pyruvate against H_2_O_2_ toxicity in a primary astrocyte culture. They demonstrated that preincubation with ethyl pyruvate (1–10 mM) provided protection, whereas preincubation with the same concentrations of sodium pyruvate had no protective effect. The same study also found that sodium pyruvate was significantly less effective than ethyl pyruvate in inducing antioxidant genes such as heme oxygenase 1 (HO-1), glutathione S-transferase (GST), and NAD(P)H:quinone oxidoreductase (NQO1) in this cell model [95]. Another study showed that ethyl pyruvate had significantly higher antioxidant activity against H_2_O_2_ and superoxide anion in chemiluminescence measurements than sodium pyruvate [55]. In addition, protective effects of ethyl pyruvate against inflammation-induced multi-organ damage have been demonstrated in animal models (see Yang et al. [3] for a review). However, in a placebo-controlled phase II study evaluating the safety and efficacy of intravenously administered ethyl pyruvate in patients undergoing cardiac surgery, ethyl pyruvate failed to demonstrate a protective effect against postoperative complications, markers of inflammation, or organ dysfunction [106]. One of the suggested reasons for the significant differences in the effects of ethyl pyruvate between animal models and human studies is the difference in the metabolism of this compound in the two groups. Specifically, ethyl pyruvate in animal plasma is hydrolysed by the enzyme carboxylesterase, releasing the pyruvate anion [107]. However, unlike most rodents, human blood plasma does not contain this enzyme [108,109]. The absence of carboxylesterase activity in human plasma, and conversely its high activity in rodent plasma, may explain the differences in the effects of ethyl pyruvate between human and rodent studies [72,107]. In fact, the activity of certain classes of esterase enzymes (including the aforementioned carboxylesterase) in human tissues differs from that in other species such as mouse, rat, rabbit, or dog. This makes it difficult to find a suitable human surrogate in esterified prodrug studies [108,109].

The safety of pyruvate therapy has been addressed in a review article by Zhou [110]. The reviewed studies of pyruvate administration in clinical settings indicated that pyruvate, when administered intravenously to patients with diabetes or liver disease, or intracoronary to patients with heart disease, is well tolerated, with no major side effects occurring during treatment. Only gastrointestinal disturbances were observed following oral administration [110]. For example, the oral administration of 15–20 g of sodium pyruvate (in single doses, three times a day for seven days) to diabetic patients caused diarrhoea. However, no gastrointestinal reactions were observed at lower doses of pyruvate (less than 10 g) [14]. Minor gastrointestinal complaints were also observed in patients with glaucoma during a 3-week period of oral pyruvate therapy at an ascending dose of 1.5 to 3 g (in combination with nicotinamide treatment). These complaints did not require intervention, however, and resolved with continued treatment [15]. In a study by Petkova et al. [13], 21 patients with chronic liver disease were administered sodium pyruvate intravenously at doses of 54–86.6 g/day for 10 days or 50–54 g/day for 15 days. The majority of patients (approximately 94.4%) tolerated the treatment well or very well, while approximately 5.5% experienced satisfactory or poor clinical tolerability (adverse effects included changes in blood pressure, pulse rate, and clinical appearance). Additionally, no adverse effects were observed when sodium pyruvate nasal spray was administered to patients infected with SARS-CoV-2 [16].

## 5. Conclusions

This review highlights the often-overlooked pyruvate molecule’s cytoprotective properties, exploring its potential to protect against environmental challenges. In vitro and in vivo studies demonstrate that during exposure to toxic chemicals and physical agents, exogenous pyruvate directly neutralises ROS (primarily H_2_O_2_), protects and stabilises mitochondrial function, increases antioxidant defences, and maintains ATP levels. Pyruvate also exhibits anti-inflammatory effects. Furthermore, a key advantage of using pyruvate as a supplement is its broad therapeutic index, which has been confirmed in clinical investigations. This makes pyruvate safe to use without risking unacceptable side effects.

However, the review also identified several areas that require further clarification in future scientific research.

1. Existing research on the effects of exogenous pyruvate in the context of physical and chemical toxicants has mainly focused on the ethyl derivative of pyruvate, particularly in animal models. As previously mentioned, however, this derivative may not be effectively converted to free pyruvate in humans. Therefore, in vivo studies using sodium pyruvate could provide a more accurate assessment of metabolism and protective effect of exogenous pyruvate, particularly at the level of individual organs.
2. In addition, consideration should be given to the potential side effects associated with the formation of toxic pyruvate dimers and the risk of hypernatraemia that may accompany the administration of higher doses of sodium pyruvate.
3. An important area of research is the role of mitochondria in the protective mechanisms of exogenous pyruvate, given their key importance in the cellular metabolism of this compound.
4. Finally, the development of stable forms of pyruvate could offer additional opportunities for the therapeutic use of this α-keto acid.

## Figures and Tables

**Table 1:** Main applications of pyruvate compounds in various fields.

Application Area	Innovative and Established Uses	References
Drug industry	Oral sodium pyruvate: possible mitigation of mitochondrial diseases in human studies (insufficient data). Inhaled sodium pyruvate: anti-inflammatory effect in pulmonary diseases (N115, a drug containing sodium pyruvate, is in pre-registration for idiopathic pulmonary fibrosis).	[1,12,16,17]
Cosmetics	Pyruvic acid: exfoliating agent in dermatology	[18]
Diet supplements	Oral calcium pyruvate, sodium pyruvate, creatine pyruvate: possible improvement in exercise performance and weight loss aid (results inconclusive).	[19,20,21]
Nutraceutical and food industry	Pyruvate, phosphoenol pyruvate: starting materials for amino acid production. Ethyl pyruvate: a flavouring agent and preservative used in the food industry.	[22,23]

**Table 2:** Summary of animal studies evaluating pyruvate effects on toxicity of physical and chemical agents.

Pyruvate Compound	Type of Toxicant	Animal Model	Effects	Proposed Mechanism (s)	References
Physical Agents					
Ethyl pyruvate 60 mg/kg, iv	heat stroke (heating chamber, 42 °C)	rats	EP mitigated the damage caused by heat stress to the liver, heart, lungs, skeletal muscle, and intestinal epithelium. Increased rat survival was observed.	■ Anti-inflammatory effects (reduction of TNFα, IL-6, NF-κB, HMGB1) ■ Induction of stress proteins (Hsp, HO-1) ■ Maintenance of autophagy	[36]
Ethyl pyruvate 40 mg/kg, ip	thermal injury (shaved dorsal skin exposed to boiling water for 12 s)	rats	EP alleviated the lung tissue damage caused by heat stress.	■ Antiapoptotic effects ■ Decreased myeloperoxidase (MPO) activity (marker of neutrophil accumulation in the lungs)	[47]
sodium pyruvate 250 mg/kg bw orally or 50 mg of 5% *w*/*w* sodium pyruvate ointment	UV-B, 280–315 nm (shaved dorsal skin exposed to UV-B for 2 min)	guinea pigs	Both oral and topical sodium pyruvate were found to have protective effects against UV-induced dermal erythema.		[33]
Pyruvate 500 mg/kg, ip	UV-B, 280–320 nm 500 J/m^2^ on dorsal skin	mice	Pyruvate protected the epidermis of mice from UV-induced DNA damage, as measured by levels of cyclobutane pyrimidine dimers.		[34]
Aromatic pyruvates (10 μM indole-3-pyruvate on dorsal skin)	UV-B, 290–320 nm 1 J/cm2 on dorsal skin	mice	Pyruvate significantly alleviated UV-B-induced erythema on the back of the skin, improved skin morphology, and prevented skin barrier damage.	■ Anti-inflammatory effects (reduction of IL-6, Cox-2) ■ Antiapoptotic effects	[32]
Ethyl pyruvate 70 mg/kg/day for 5 days, ip	Ionizing radiation (gamma) Total body dose of 9.75 Gy	mice	EP has increased the survival rate of animals exposed to radiation		[37]
Ethyl pyruvate 100 mg/kg/day for 28 days ip	Ionizing radiation (X-rays) Whole lung irradiation, 16 Gy	mice	EP partially relieved radiation-induced lung injury	■ Anti-inflammatory effects (reduction of IL-1β and IL-6, HMGB1, TGF-β1)	[38]
**Chemical Agents**					
Ethyl pyruvate 50, 100, 200 mg/kg/day for 14 days, orally	AlCl_3_ 50 mg/kg/day for 28 days, ip	rats	EP attenuated the histopathological changes induced by AlCl_3_ in the cerebral cortex (reduced β-amyloid plaque deposition). It also improved cognitive function.	■ Antioxidant effects (reduction of MDA, increase of SOD, GSH) ■ Inhibition of pro-inflammatory Toll-like receptor 4 overexpression	[43]
Ethyl pyruvate 40 mg/kg/day for 35 days, ip	Phenylhydrazine 8 mg followed by 6 mg per 100 g every 48 h for 35 days, ip	mice	Mitigation of phenylhydrazine-induced sperm damage (number, morphology, motility, viability)	■ Antioxidant effects (reduction of MDA in testicular tissue)	[44]
Ethyl pyruvate 50 and 100 mg, ip	Paracetamol 400 mg/kg, ip	mice	EP attenuated paracetamol-induced histopathological liver damage	■ No antioxidant effects (marker: hepatic nitrotyrosine formation)	[39]
Etyl pyruvate 40 mg/kg/day for 35 days, ip	Cyclophosphamide 15 mg/kg/week for 35 days, ip	mice	EP reduced the histopathological damage to the testes caused by cyclophosphamide.	■ No antioxidant effects (marker: serum TAC)	[48]
Ethyl pyruvate 50 mg/kg/day for 14 days, ip	Amikacin 600 mg/kg/day for 14 days, im	rats	EP alleviated the damage to hearing caused by amikacin	■ Antioxidant effects (reduction of MDA, increase of TAC in cochlea tissue) ■ Anti-inflammatory effects (reduction of IL-1β and IL-6, TNF-α)	[49]
Ethyl pyruvate 50 mg/kg for 2 weeks, ip	Ketamine 25 mg/kg for 2 weeks ip	mice	EP alleviated ketamine-induced cognitive impairment	■ Antioxidant effects (reduction of MDA in brain tissue)	[50]
Ethyl pyruvate 40 mg/kg (three doses), ip	Carbon tetrachloride (CCl_4_) 1.6 g/kg	rats	EP attenuated CCl_4_-induced histopathological liver damage	■ Antioxidant effects (normalisation of markers of oxidative stress) ■ Anti-apoptotic effects	[45]
Ethyl pyruvate 40 mg/kg/day for 30 days, ip	Methotrexate 20 mg/kg once per week for 30 days, ip	mice	EP attenuated the negative effects of methotrexate on male mouse fertility (marker: in vitro fertilisation rate and embryonic development)	■ Antioxidant effects (reduction of MDA, increase of CAT in testicular tissue)	[40]
Ethyl pyruvate 40 mg/kg/day for 35 days ip	Sodium cyanide 2 mg/kg ip	mice	EP attenuated NaCN-induced histopathological changes in the testes and the negative effects of NaCN on fertility in male mice (markers: in vitro fertilisation rate and embryonic development).	■ Antioxidant effects (reduction of serum MDA)	[51]
Ethyl pyruvate (50 mg/kg for 5 days, ip)	Cisplatin (5 mg/kg for 5 days ip)	rats	EP attenuated cisplatin-induced histopathological changes in the kidney	■ Antioxidant effects (reduction in renal MDA, increase in total serum antioxidant status) ■ Anti-inflammatory effects (reduced inflammatory cell infiltration)	[52]
ethyl pyruvate 40 mg/kg/day for up to 6 weeks, ip	CCl_4_ (CCl_4_:olive oil = 1:1) 3 mL/kg, twice a week for up to 6 weeks, sc	rats	EP attenuated the histopathological changes and liver fibrosis induced by CCl_4_	■ Anti-inflammatory effects (reduction of HMGB1, IL-6, TNF-α in liver) ■ Reduced activation of hepatic stellate cells ■ Inhibition of overexpression of the pro-inflammatory Toll-like receptor 4/NF-κB pathway	[46]
Ethyl pyruvate 100 mg/kg for 15 days, orally	Ethanol 50% solution (10 mL) once, ip and 4 g/kg/day (52%) for 4 weeks, orally	mice	EP alleviated alcohol-induced liver histopathological changes	■ Anti-inflammatory (reduction of pro-inflammatory IL-6 and TNF-α in the liver) ■ Antioxidant activity (reduction of MDA) ■ Inhibition of Nrf2/very low density lipoprotein receptor (VLDLR) pathway overexpression	[42]
Ethyl pyruvate 50 mg/kg for 6 days, ip	Paclitaxel (Taxol) 2 mg/kg, every other day, ip	rats	EP did not significantly affect paclitaxel-induced allodynia		[41]
Ethyl pyruvate 40 mg/kg, ip	phosgene gas 10% for 1 min in whole-body chamber	rats	EP alleviated phosgene gas-induced pulmonary oedema	■ Anti-inflammatory effects (reduction of pro-inflammatory NO and PGE2 in the lungs) ■ Inhibition of MAPK/COX-2 and MAPK/iNOS pathways	[53]
Ethyl pyruvate 40 mg/kg every 8 h for up to 48 h, ip	Paracetamol (APAP) 350 mg/kg, ip	mice	Early phase in APAP overdose: EP attenuated paracetamol-induced histopathological changes in the liver Late phase in APAP overdose: EP increased paracetamol-induced liver histopathological changes		[54]
0.3–3% EP in drinking burette for 10 weeks	10% ethanol solution in drinking burette for 10 weeks	rats	EP protected white blood cells from alcohol-induced damage	■ Reduction of DNA damage in lymphocytes ■ Anti-inflammatory effects (reduction of superoxide anion production by monocytes)	[55]
Pyruvate 500 mg/kg, sc	ethanol (2.5 g/kg), sc	mice	pyruvate attenuated ethanol-induced neurodegenerative changes in the brain	■ Anti-apoptotic effects	[35]

Abbreviations: CAT, catalase; COX-2, cyclooxygenase 2; EP, ethyl pyruvate; GSH, reduced glutathione; HMGB1, high mobility group box 1; HO-1, heme oxygenase-1; Hsp, heat shock protein; IL, interleukin; im, intramuscularly; iNOS, inducible nitric oxide synthase; ip, intraperitoneally; iv, intravenously; MAPK, mitogen-activated protein kinases; MDA, malondialdehyde; NF-κB, nuclear factor kappa B; NO, nitric oxide; PGE2, prostaglandin E2; sc, subcutaneously; SOD, superoxide dismutase; TAC, total antioxidant capacity; TNF, tumour necrosis factor; UV, ultraviolet radiation.

**Table 3:** Summary of cell culture studies evaluating pyruvate effects on toxicity of physical and chemical agents.

Pyruvate Compound	Type of Toxicant	Cell Culture Model	Effects	Proposed Mechanism (s)	References
Physical Agents					
Sodium pyruvate 1, 3, 5, 10 mM	Light-induced damage 15,000 lux light for 5 h	Murine photoreceptor-derived 66lW cells	SP improved morphology and significantly reduced light-induced cell death	■ Reduced H_2_O_2_ production ■ Increased ATP production	[26]
Ethyl pyruvate 10 mM	Ionizing radiation (gamma), 0–8 Gy	32Dcl3 mouse hematopoietic progenitor cell line	EP increased survival of irradiated cells	■ Anti-apoptotic effects	[37]
Sodium pyruvate 1 mM	UV-A, 340–420 nm single UV-A dose or 3 times/day (for 4 days)	Primary human skin fibroblasts		■ Reduced ROS generation and slight reduction in 8-OHdG levels ■ Reduced expression of matrix metalloproteases (enzymes associated with photo-aging)	[56]
Ethyl pyruvate 0.2–5 μM	Ionizing radiation (X-ray), 0–8 Gy	RAW264.7 macrophages, human bronchial epithelial cells	EP increased survival of irradiated cells	■ EP reduced extracellular HMGB1 release	[38]
Pyruvate 1000 ng intracutaneously	UV-B, 280–320 nm, 500 J/m2	Human skin	Reduced levels of cyclobutane pyrimidine dimer (DNA damage marker)		[34]
**Chemical Agents**					
Ethyl pyruvate 5, 10, 20, 40 mM	AlCl_3_ 1250 μM	Primary neuron–glial mixed cells	EP significantly reduced AlCl_3_-induced cell death		[43]
Sodium pyruvate 4.5 mM	VOSO_4_ 100 μM	Chinese hamster ovary K1 cells	SP improved morphology and significantly increased cell viability limited by VOSO_4_	■ Restoration of the correct mitochondrial membrane potential	[61]
Sodium pyruvate 10 mM	Oxyradicals species (xanthine oxidase-induced)	Neural retina (from mice)	SP prevented ROS-induced inhibition of glycolysis and lactate formation in the retina	■ Increase in the NAD+/NADH ratio	[65]
Ethyl pyruvate 0.01–1 mM	a reactive metabolite of APAP, N-acetyl-p-benzoquinone imine (NAPQI), 0.4 mM	human hepatoma cell line (HEPG2 cells)	EP improved cell viability limited by NAPQI	■ Anti-apoptotic and anti-necrotic effect	[39]
Sodium pyruvate 2.5–25 mM	H_2_O_2_ (0.128–32.768 mM)	Human fibroblasts (Hs27 cell line)	SP improved cell viability limited by H_2_O_2_	■ Reduced ROS generation ■ Increase in mitochondrial membrane potential	[59]
Sodium pyruvate 8 mM	CdCl_2_ 10, 30 and 100 μM	Murine hippocampal HT-22 cells	SP improved cell viability limited by CdCl_2_	■ Increased glycolysis ■ Decreased ROS generation ■ Increase in mitochondrial membrane potential ■ Anti-apoptotic and anti-necrotic activity	[60]
Ethyl pyruvate (1–15 mM)	ZnSO_4_ (40 or 400 μM)	Primary cortical cultures from mouse	EP improved cell viability limited by ZnSO_4_	■ NAD replenishment ■ Reduced ROS generation ■ ability to chelate Zn2+ by EP	[62]
Pyruvate 5 mM	ZnCl_2_ (40, 200 or 300 μM depending on the measured endpoint)	Mix mouse cortical and pure hippocampal neuronal cells	Pyruvate reduced ZnCl_2_-induced cell death	■ Pyruvate increases citrate and isocitrate levels in cells, which chelate Zn2+, preventing Zn cytotoxicity.	[58]
Pyruvate 10 mM	*p*-aminophenol (0.1–0.5 mM)	Renal cortical slices from rats	Pyruvate reduced *p*-aminophenol-induced cell death	■ Increase in adenine nucleotides (ATP, ADP, AMP) levels ■ Increase in intracellular GSH levels through NADPH-mediated recycling of GSSG to GSH	[64]
Pyruvate 1 mM	H_2_O_2_ 10–1000 μM	Primary osteoblasts from rats	Pyruvate reduced H_2_O_2_-induced cell death	■ The important contribution of pyruvate transporters (monocarboxylate transporters) to its cytoprotective properties	[66]
Ethyl pyruvate 2.5 μM	Organic dust	human bronchial epithelial cells (BEAS-2B)		■ Reduced expression of HMGB1 in the cytoplasm ■ Reduced levels of pro-inflammatory factors (NF-κB, GM-CSF, IL-1β)	[57]
Ethyl pyruvate (2, 5 or 10μM)	phosgene 400 ppm	RAW 264.7 macrophages		■ Reduced levels of pro-inflammatory mediators, i.e., prostaglandin E2 and NO	[53]
Sodium pyruvate (0.5, 1, 2 mM)	Cigarette smoke extract	A549 (human lung carcinoma epithelial cells) and BEAS-2B (bronchial epithelial cells) cell lines	Pyruvate improved cell viability limited by cigarette smoke	■ Reduced ROS generation, MDA level, and increased GSH level ■ Increased mitochondrial membrane potential ■ Reduced ferroptosis via activating the GPx4/Nrf2 pathway ■ Reduced levels of pro-inflammatory factors (COX2, IL-8, TNF)	[63]

Abbreviations: COX2, cyclooxygenase 2; EP, ethyl pyruvate; GM-CSF, granulocyte macrophage colony stimulating factor; GPx4, glutathione peroxidase 4; GSH, reduced glutathione; H_2_O_2_, hydrogen peroxide; HMGB1, high mobility group box 1; IL, interleukin; MDA, malondialdehyde; NF-κB, nuclear factor kappa B; NO, nitric oxide; Nrf2, nuclear factor E2-related factor 2; 8-OHdG, 8-hydroxydeoxyguanosine; ROS, reactive oxygen species; SP, sodium pyruvate; TNF, tumour necrosis factor; UV, ultraviolet radiation.
